# Taking Another Look: Thoughts on Behavioral Symptoms in Dementia and Their Measurement

**DOI:** 10.3390/healthcare6040126

**Published:** 2018-10-22

**Authors:** Diana Lynn Woods, Kathleen Buckwalter

**Affiliations:** 1 School of Nursing, Azusa Pacific University, Azusa, CA 91702, USA; 2 College of Nursing, University of Iowa, Iowa City, IA 52242, USA; kathleen-buckwalter@uiowa.edu

**Keywords:** reconceptualization, behavioral symptoms, measurement, clustering

## Abstract

This article proposes taking another look at behavioral symptoms of dementia (BSDs) both from a theoretical perspective that informs research and practice and from a measurement perspective. We discuss why this rethinking of behaviors impacts current models of care and our ability to better detect outcomes from interventions. We propose that BSDs be viewed from a pattern perspective and provide some suggestions for how to identify and measure these patterns that can influence the timing and type of intervention. Evidence suggests that BSDs are complex, sequential, patterned clusters of behavior recurring repeatedly in the same individual and escalate significantly without timely intervention. However, BSDs are frequently viewed as separate behaviors rather than patterns or clusters of behaviors, a view that affects current research questions as well as the choice, timing, and outcomes of interventions. These symptoms cause immense distress to persons with the disease and their caregivers, trigger hospitalizations and nursing home placement, and are associated with increased care costs. Despite their universality and that symptoms manifest across disease etiologies and stages, behaviors tend to be underrecognized, undertreated, and overmanaged by pharmacological treatments that may pose more harm than benefit.

## 1. Introduction

Evidence suggests that behavioral symptoms of dementia (BSDs) are complex, sequential, patterned clusters of behavior recurring repeatedly in the same individual and escalate significantly without timely intervention [1]. However, BSDs are frequently viewed as separate behaviors rather than patterns or clusters of behaviors, a view that affects current research questions as well as the choice, timing, and outcomes of interventions. Behavioral symptoms are a hallmark of dementia, representing a heterogeneous group of non-cognitive manifestations [2], commonly occurring in most persons with dementia and across disease stages and etiologies. These symptoms are often more devastating than the cognitive deficits and are frequently associated with poorer quality of life, caregiver distress, more time in caregiving, and increased hospitalizations and nursing home placements.

Despite their prevalence and negative consequences for individuals, families, and care systems (IFCS), behavioral symptoms remain under-detected, undertreated, and overmanaged by pharmacological treatments that may cause more harm than benefit and do not address symptoms most problematic to IFCS. Although nonpharmacologic treatments are promising, they continue to be underutilized.

This paper argues for rethinking how we conceptualize, define, and measure behavioral symptoms of dementia in terms of clusters and patterns, as opposed to single behaviors. This reconceptualization may significantly impact current models of care as well as the ability to evaluate treatment outcomes.

## 2. Current Thinking about Behavioral Symptoms

Six main foci are used to briefly discuss earlier and current conceptualizations of behavioral symptoms: early definitions and views of origin, disciplinary traditions, biomedical views, behavior as a form of communication, the Dementia Initiative perspective, and Kitwood’s person-centered, personhood-oriented view [3].

The authors acknowledge that there are a variety of other conceptualizations, such as the Kales, Gitlin, and Lykestos framework used in a recent scoping review of the evidence base for determinants of selected individual BSDs including agitation, apathy, psychosis, depression, and aggression [4].

The intent of this section, however, is only to highlight selected prominent approaches to BSDs that provide background information we regard as foundational to rethinking behaviors as patterns or clusters, rather than to present an exhaustive or systematic review that evaluates the merits of each approach or favors one conceptualization over another. The way one conceptualizes BSDs influences the way in which behaviors are examined and measured, as well as the type of interventions developed and the manner in which care is provided.

How behavioral symptoms are conceptualized and defined has changed over time, and still varies today. An early definition of the term “behavioral disturbance” referred to “a behavioral or psychological syndrome or a pattern associated with subjective distress, functional disability or impaired interactions with others in the environment” [5], which evolved to behavioral and psychological symptoms of dementia (BPSDs) [6]. BSDs affect almost all persons with dementia (PWDs) at some point over the course of their illness [7], tend to fluctuate, and seldom occur in isolation [8]. Most perspectives, as reflected in the early definition above, are deficit-oriented, viewing BSDs in a negative light, as something to be “fixed” or eliminated. More recently, investigators have argued for understanding the meaning of actions and behaviors rather than pathologizing them [9].

Definitions also vary according to disciplinary traditions. Recognizing that dementia is not a disease of cognition alone, Donnelly [10] referred to BSDs as “non-cognitive neuropsychiatric symptoms” (NPSs). Included in this conceptualization are behaviors such as aggression, agitation, depression, anxiety, delusions, hallucinations, apathy and disinhibition, sleep disturbances, and executive dysfunction [2,11].

Biomedical models emphasize that BSDs arise from measurable anatomical and biochemical changes such as the association of agitation with bilateral orbitofrontal and left anterior cingulated tangles [12]. Moreover, from this perspective, neurochemical changes, alterations in neural structure and genetics, are emphasized. For example, using neuroimaging, Poulin and colleagues [13] found that aggressive behavior was associated with amygdala atrophy, while Berlow and colleagues [14] found that anxiety, sleep disorders, and aberrant motor behavior was associated with increased white matter hyperintensities. Furthermore, Boronni and colleagues [15] found sleep disturbances and delusions associated with catechol-O-methyl transferase (COMT) in the Dopamine pathway. Suffice it to say that, while imaging has helped to potentially locate neuroanatomical areas associated with behavioral symptoms, they do not fully explain the origin or clusters of behaviors.

In contrast to the biomedical conceptualization of BSDs that emphasizes the role of neurochemical, neuropathological, and genetic factors [7], multidisciplinary participants in the Dementia Initiative [16] preferred the term “behavioral expressions,” which has fewer paternalistic connotations, a less negative orientation, and favors a person-centered approach to dementia care. This term also reflects an understanding that behaviors may be expressions of unmet needs that a person with dementia is unable to communicate [17,18]. An earlier but related conceptualization [3] sought to keep the PWD at the forefront of care, fostering their wellbeing rather than considering BSDs as a negative consequence of dementia. From this perspective, caregivers strive to respectfully provide meaningful activities focusing on retained strengths and interests rather than losses.

In their recent scoping review, Kolanowski and colleagues noted, “Nursing has a rich history of conceptualizing BPSD as expressions of unmet needs within frameworks, where a number of pathophysiologic, psychological, and environmental determinants are hypothesized to underlie BPSD” that have guided research [4]. They caution, however, that intervention studies based on these frameworks have seldom found large effect sizes. One early conceptual model of care for persons with dementia, the Progressively Lowered Stress Threshold Model, developed by a nurse clinician and scientist, noted that all behavior has meaning [18] and that behavior is a form of communication. Further, behaviors may arise when levels of stimuli, both external such as large groups and loud noises, and internal, such as pain or hunger, exceed the person with dementia’s ability to tolerate stress. Care management strategies are therefore aimed at knowing what environments, factors or people escalate individual stress and keeping those factors in balance, and intervening before they may be disturbing to the person with dementia. Experts have long agreed that the inability of PWDs to express what they want to say or to verbally communicate concepts such as pain, loneliness, and boredom can lead to frustration, feeling upset, and agitation that is often communicated by body language, gestures, actions, and behaviors [19].

The Need-Driven Dementia-Compromised Behavioral Model (NDB), also developed from a nursing perspective and set forth by Algase and colleagues [17], posits the need to change our view of behaviors as “problematic” or “disturbing” to one of understandable expression of unmet needs, reflecting the interaction of stable background factors and environmental triggers. The NDB Model was extended by Kovach, Noonan, Schlidt, and Wells [20] to include consequences of need-driven behaviors in PWDs, or expressing needs behaviorally rather than verbally. Inherent in these nursing approaches to care of PWDs is also the need to support and educate caregivers, both formal and informal, and to treat them and the person with dementia as respected adults.

## 3. Etiology of Behaviors

Conceptualization of BSDs depends on the perspective of the origin of these behaviors. Many agree that cognitive impairment alone does not explain the etiology of behaviors. A variety of factors are believed to contribute to BSDs [4], including changes in the brain (biological factors), psychosocial factors such as high levels of premorbid anxiety or depression, and situational stressors such as pain, fatigue, and confusing stimuli [7,21]. As patients with dementia experience heightened vulnerability to their environment, behavioral symptoms may result from the confluence of multiple, some potentially modifiable, interacting factors including internal (e.g., pain, fear), [22] or external (e.g., over-stimulating environment, complex caregiver communications) features [23].

Single BSDs such as wandering [24,25], aggressive behavior [26,27], and disruptive vocalization [28,29] have been extensively studied. Early work by Hall and Buckwalter [18], based on clinical observations, suggested that BSDs were related to a progressively lowered stress threshold (PLST). Other explanations for BSDs have included the idea that behaviors may be driven by an individual’s inability to express a need (or Need Driven Behavior, NDB) [17], resulting in behaviors such as restlessness, problematic vocalization, and wandering. More recent work by Whall et al. [26] and Algase et al. [25] examined factors underlying aggressive behavior (AB) and wandering [30,31]. Whall et al. found that AB was related to three factors: gender, stage of dementia as measured by the Mini Mental State Exam (MMSE) [32], and the degree of pre-morbid personality agreeableness. Algase et al. found that wandering differs from restlessness and is potentially related to a disordered frontal lobe. Environmental stressors may act as triggers in vulnerable individuals. All of these models have implicit/explicit characterizations of BSDs that subsequently influence research and practice.

## 4. Conceptualizing and Characterizing Patterns in BSDs/Behaviors Occurring in Clusters

Although numerous investigators have studied BSDs and have viewed them from a variety of perspectives as noted earlier [24,25,26,29,33,34,35,36,37], there remains little understanding of its complex organization. Research suggests that more than a third (38%) of PWDs have purposeless activity such as restlessness that is repetitive in nature, and 29–45% [29,38] exhibit problematic vocalization [39]. Although only a third (32%) of behaviors of behaviors occur alone, more than double that amount, or 66.4%, co-occur [15], and 18% co-occur within the same hour [39]. These behaviors can have significant consequences; for example, restlessness is strongly associated with reduced caregiver quality of life and premature nursing home (NH) placement [40].

Non-pharmacological interventions are currently recommended for BSDs [41] based in part on the premise that these behaviors arise as a consequence of the PWD’s response to stress [18]. Two main approaches to reducing stress through nonpharmacological means are offered. The first assumes that stress can be relieved by relaxation and engendering a relaxation response; the second focuses on triggers. Both of these approaches address internal (pain, fear, sleep) and external factors (the environment and complex caregiver communication). First, researchers have studied the effects of treatments believed to produce relaxation, such as individualized music [42] calming touch [43], and aromatherapy [44,45]. Overall these interventions have demonstrated small to moderate effects with a short or unknown duration of action [46,47]. In addition, there is great within-group variation in response to treatment, a finding that has remained unexplained.

The second approach has focused on identifying BSD triggers, so that the intervention can address them. This research was guided by the assumption that given their decreased cognitive abilities, PWDs have difficulty processing environmental stimuli, including those arising from social interactions, and that if triggers can be identified and blocked, BSDs will be reduced [18,48,49]. For example, Radgneskog et al. [50] found that an increased number of BSDs was related to loss of control over a situation and loss of independence in addition to environmental noise and invasion of personal space.

The quality of interactions with people has also been studied as a trigger for BSDs, with equivocal results. For example, studies have shown that the likelihood of BSD onset during periods of verbal interaction with staff is reduced for some residents but increased for others and that the frequency and quality of social interactions with other residents, family, and visitors is negatively correlated with problematic vocalizations [51,52]. Kolanowski and Litaker [53] found a positive relationship between the number of social interactions and the number of BSDs, suggesting that an inappropriate amount of interaction might elicit BSDs, because too little or too much interaction may frustrate PWDs, thereby leading to further BSDs [51].

Herman and Williams [54] and Williams and Herman [23] examined the effect of elderspeak, an intergenerational communication style between staff and residents frequently associated with demeaning language. They found a significant co-occurrence between the use of elderspeak and resistiveness to care. While somewhat promising, these and similar studies have been limited by small samples, small effect sizes, and large intergroup variability. Hence, while the association between triggers and BSDs has been examined over the past 40 years, understanding the contribution of various triggers to BSDs remains elusive.

Recurrences of BSDs vary considerably within and between individuals. This variability is a major methodological block to the establishment of an analytic technique to reliably assess the severity, change, and temporality of BSDs [55,56]. Without the ability to meaningfully characterize BSDs, the relationship between these complex behavioral patterns and other factors, such as the environment or personality traits, remain difficult to detect reliably. Further, a recent systematic review of the literature looked at the incidence and persistence of 11 different BSDs (called BPSD in the published article), and found differences in the longitudinal courses of different behaviors and symptoms [57].

Despite numerous studies of interventions to treat BSDs, no approaches have emerged as robust treatments. Treatment effects are small, and individual response patterns vary [58]. Some speculate [59] that the development of effective interventions is compromised by measurement problems [29]. This problem is related to how we have conceptualized BSDs, contributing to our inability to quantify BSDs in ways that allow for detection of subtle behavior changes that might signify intervention responses.

The way we conceptualize behavioral symptoms is important as this continues to influence models of care, research methodologies and to guide clinical practice. This understanding has led to a focus on the quantification of frequency and severity of symptoms, devaluing the context in which a behavior occurs, particularly as we now appreciate the important role environmental factors can play in contributing to and reducing or resolving BSDs. Similarly, enduring habits, personality traits, life experiences, co-morbid conditions, and their treatment can influence what types of behaviors occur [60].

## 5. Measurement Issues/Problems

Accurate measurement of behaviors is essential for clinicians and researchers alike for a number of reasons. They must be able to track dementia progression, monitor the effectiveness of pharmacologic and nonpharmacologic interventions, and examine correlates of caregiver outcomes such as burden and coping. The importance of reliably capturing behaviors is evident, and yet there is no “gold standard” measure or methodology for operationalizing behaviors, despite the fact that for more than two decades clinicians and researchers have argued for a “brief, conceptually and psychometrically sound method for assessing behavioral problems in patients with dementia” [61] (p. 622). Years ago, Davis and colleagues [62] noted that variations in definition and measurement across studies have hampered efforts to draw meaningful conclusions about behaviors in dementia. Comments that remain true today. As van Derlinde and colleagues note [63] (p. 95), “Better measurements of these symptoms are needed to improve knowledge of their prevalence, associations and management.” This need is echoed by Kolanowski et al. [4] (p. 516) who argue that the wide variety of symptoms associated with BSDs “are often measured inconsistently and imprecisely as one construct.”

The ABC model of behavior (antecedent–behavior–consequence) [48,49,64] describes BSDs as single troublesome, or unsafe behaviors (“target behaviors”) that usually emerge following a stressful event or stimuli. This perspective has heavily influenced BSD research. Under this single behavior approach, some researchers [34,65] have conceptualized BSDs as comprised of clusters or subtypes of disturbing or potentially dangerous behaviors. The single-behavior approach to studying BSDs has been used extensively including research on wandering [24,25,33], aggressive behavior [27], and problematic vocalizations (yelling) [28,29,66,67].

Most measures of BSDs, using the single behavior approach, rely on frequency (presence or absence) of the target behavior. The Cohen Mansfield Agitation Inventory (CMAI) [35] is the most widely used instrument for measuring BSDs. Moreover, this approach is not sensitive to the subtlety and discrete data that is needed to potentially characterize behaviors. Rarely considered in this type of measurement is that the absence of a behavior can also mean (as frequently happens with use of psychotropic drugs), that the target behavior is absent, but so are functional behaviors, (i.e., the PWD is somnambulant). Another factor complicating measurement using the single behavior approach is that data are often elicited using proxy reporting (family caregivers and/or nursing personnel), using scales such as the BEHAVE-AD [68]. While there is efficiency in this approach in terms of time, it is fraught with problems since caregivers’ recall of BSDs is strongly influenced by their psychological state and needs [69].

Using a Functional Analysis Checklist (FAC), a checklist that uses the ABC model of behavior, James and colleagues [70] assessed the ability of 76 staff members to indicate situations and settings that triggered challenging behaviors. Each pair of staff rated antecedents. Results indicated that staff agreement was poor related to what may be triggering these challenging behaviors. James and colleagues concluded that training about systematic observation and reporting on the nature of behaviors may result in higher agreement scores. Another issue may be the reliance on a priori assumptions, such as are evident in the ABC model, rather than using observed quantitative data and an algorithmic approach to assessing the clustering of behaviors.

Two major sources of error are possible: caregiver exaggeration, where the caregiver reports an increased number of events, and caregiver denial, where the few or benign behavior problems are not reported [71,72]. In addition, caregivers’ reports can be unstable from hour to hour and day to day, changing as their own needs change. Another problem is that scales such as the BEHAVE-AD take a global approach to measurement. Because they rely on recall, behaviors are “lumped” together over a specified time period (e.g., yesterday, or in the last week), even though studies show that BSDs have temporal patterns [73,74,75], meaning that they vary in more or less predictable ways throughout the day. Lumping leads to a lack of specificity with the loss of temporal variation. In addition, the recall of one behavior at one time may be considered sufficient for a global categorization of “displaying BSDs”, but not for determining the temporal or individual variation.

Researchers have long recognized these limitations. For example, Cohen-Mansfield further refined the CMAI by developing the Agitated Behavior Mapping Instrument (ABMI) for use with direct observation [35]. While an improvement over informant reporting, this scale measures only the frequency of a behavior and not the intensity, remaining focused on single target behaviors [69]. That BSDs are characterized by intensity as well as frequency is rarely considered. Intensity is defined as the troublesomeness of the behavior. Yelling, for example, is more troublesome than mumbling or picking at the bedclothes. The only scales to date that rate both the frequency and the intensity of BSDs using direct observation are the Agitated Behavior Rating Scale (ABRS) [76] and the refined version of the Modified Agitated Behavior Rating Scale (mABRS) [77]. For example, with vocalization, mumbling is scored at an intensity of 1, while yelling is scored at an intensity of 3. Direct observation allows for specificity about the type, intensity, and frequency of behavior and permits exploration of temporal variation.

While current conceptualizations of BSDs include the concept of behavioral patterns, measuring these patterns remains challenging [78]. Two types of patterns have been considered: temporal patterns and patterns of escalation. Both patterns have been acknowledged and examined for the past several years using a variety of methods in an attempt to determine the time of peak behavior, albeit using aggregated data, and instruments designed to be used in cross-sectional studies [69]. Previous examination has been limited by the statistical methods and computer technology available as well as the breadth of accessible data.

Patterns of escalation describe an increase in (usually frequency and less commonly intensity) a specific behavior. For example, vocalization may progress from repeating a word to screaming. Several researchers have described a sequence of behavioral escalation that has clear starting and ending points, calm behavior progressing to violence. Behavior that follows an “ideal” sequence from verbal agitation escalating to verbally aggressive behavior suggests a linear pattern. The reverse, de-escalation, is assumed but rarely described. Less severe/intense behaviors are placed lower on the continuum, while more severe/intense behaviors are higher [1]. Escalation can also proceed from one behavioral category to another, suggesting that these patterns are more complex, increasing in variability while co-occurring [78,79].

Temporality, the period of time within which a behavior changes, can be demonstrated in the case of vocalization. This behavior may begin as repetitive mumbling and escalate to louder calling, then yelling within a period of 20–40 min [1]. Viewed from this perspective, a model of escalation predicts that PWDs initially demonstrate low-intensity BSDs, which, when they continue over time, may escalate to a more severe behavior. However, in contrast to the “ideal” escalation/de-escalation pattern, Woods et al. [1] examined patterns of escalation of BSDs using videotaped data, finding that the “ideal” pattern was rarely observed. Rather, patterns moved back and forth between behavioral categories rather than escalating within categories in a linear fashion. This suggests that patterns escalate in frequency and intensity as well as complexity (i.e., an increase in variability among co-occurring behaviors).

To further confound measurement issues there is a great deal of intra- and interindividual variability in the expression and frequency of behaviors among persons with dementia. Certainly behaviors are not consistently expressed by all PWDs and commonly fluctuate over the course of the illness as well as during the day (diurnal variation). Thus, while a particular instrument may capture behaviors in the early disease stages, it may not be valid later on in the disease or be sufficiently sensitive to clinical change. For example, in the early stages, personality changes, apathy, decreased capability to manage instrumental activities of daily living and irritability are common. As the dementia progresses, behaviors often become more disruptive and psychiatric symptoms may emerge, including aggression, wandering, paranoia, hallucinations, and delusions. By the late or final stages of the illness, PWDs are frequently bedbound and too debilitated to express many of the aforementioned behaviors. Bharucha and colleagues [80] suggest that it is particularly difficult to capture low-frequency high-impact behaviors over time. Other factors that can complicate the measurement of behaviors include bias in raters and scoring, a lack of bench-marking studies, construct slippage, and shifting domains [62].

Other measurement issues include attempts to integrate multiple, distinct constructs into one measure. For example, early conceptually challenged scales measured not only behaviors but also activities of daily living and cognition [80]. Further, many symptoms may overlap in conditions such as depression and apathy, agitation, and aggression.

The validity of instruments that rely on responses from the person with dementia may be compromised by their sensory loss (visual/hearing) or perceptual and cognitive deficits [80]. Other tools for which data is provided by proxy informants (e.g., clinical staff and family caregivers) can be affected by factors such as inadequate training in assessment or devaluing of the importance of careful documentation of behaviors [80]. Their own stress levels and emotional health, as well as their relationship to the person with dementia, may also affect the soundness of caregiver evaluations.

## 6. Analysis Issues/Problems

Studying patterns of BSDs is hampered by measurement as well as the analysis strategies used. In the study of temporal patterns, for example, to establish peak times of BSDs, single behaviors or several behaviors tend to be summed, aggregated, and analyzed using ANOVA for repeated measures, thus limiting the ability to detect clustered behavior patterns across time. However, data show [24,25,29,33,75,79] that recurrences of BSDs vary considerably within and between individuals. While data aggregation may demonstrate differences in response between groups, individual variation within subjects across time is lost.

Measuring individual variability may be key to the timing of interventions [55,56] and to answering the question: will this intervention work for this person? [81]. Another method used extensively to determine behavioral complexity and temporal patterns using direct observational data is lag sequential analysis (LSA) [82].

This analytic method examines the probability that a behavior will be followed by another behavior of interest and has been used to detect behavioral recurrence and temporal patterns such as those seen in persons with self-injurious behavior (SIB) [83] in persons with disabilities [36] and to a limited extent in persons with dementia exhibiting BSDs [1]. While this type of analysis allows examination of both the persistence of a behavior (behavior remaining the same within a period of time) and transition, or a change from one state (intensity) to another, a major limitation is the assumption that a temporal window can be specified, for example that the behavior occurs every 20 min. Any behavior that does not fall into this temporal window will be missed.

## 7. Rethinking BSDs

Given the acknowledged problems with measurement, the authors propose that BSDs, rather than always being single events, are complex, non-sequential, non-random, patterned clusters of behavior that recur repeatedly in the same person [1,59,78]. These behavior patterns vary by the number of clusters in a specific time period, the level of intensity of behaviors contained in the clusters, and the number of different clusters displayed in a specific time period. We conceptualize escalation as behaviors becoming more frequent, but also becoming more complex (increased in variability among co-occurring behaviors) and more intense (more troublesome) over time [1]. These patterned clusters of behavior may be linked together in predictable ways. As recently noted by Kovach in an editorial commenting on gerontological nursing research trends and needs [84] (p. 228), “When symptoms co-occur or occur in clusters, there may be important interactions and influences on health and the treatment needed.”

Therefore, escalation involves behavioral chains that begin with benign behaviors and culminate in the target behavior that can be very disruptive to family and staff caregivers. For example, particularly troublesome behaviors like yelling may be accompanied by benign behaviors (e.g., fidgeting and tapping) that may actually be part of a pattern of escalation. One reason for the failure of studies of BSD triggers to have definitive results may be that measurement begins at the point when the target behavior (e.g., yelling) occurs, although a trigger may have precipitated a behavioral chain that begins with the appearance of more benign behaviors and escalates to the target behavior. Other distinguished nurse scientists have come to this conclusion in other clinical populations. For example, Woods and colleagues [85] utilized more innovative analytic techniques, such as Group Based Trajectory Modelling and Latent Class Analysis, to elucidate how menopausal symptoms cluster and are associated with specific single nucleotide polymorphisms (SNPs) [86].

Current conceptualizations of BSDs, bound by disciplinary perspectives, have been somewhat limiting to date. As emphasized earlier, the way we conceptualize behavioral symptoms is important as this continues to influence models of care and research methodologies and to guide clinical practice. They have also led to measures that focus on the quantification of frequency and severity, devaluing the context in which a behavior occurs. The latter is particularly unfortunate as both research findings and theoretical developments have noted the powerful role environmental factors can play in contributing to BSDs. When such measures are used in a supportive manner, and consider the context, they may reduce and resolve behaviors. Similarly, long-standing habits, personality traits, life experiences, co-morbid conditions, and their treatment can also influence what types of behaviors occur [60]. For these reasons, we need to reconceptualize behavioral symptoms. This reconceptualization is germane to models of care because the models that we use, whether implicit or explicit, profoundly affect our practice.

Research outcomes that are more sensitive and that enable us to target us to target interventions more precisely are needed. The more precise we are in describing the phenomenon, the more precise and timely the intervention, thus leading to improved clinical outcomes. This need has also been recognized by Kolanowski et al. [4], who support more funding for the development of measures that will have more precision to capture the characteristics of BSDs, attending to the context in which those behaviors occur.

## 8. Alternative Measurement Strategy: Principles of Measurement

Consistent with our conceptualization of BSDs, we suggest using a pattern recognition software strategy such as THEME™ software [87] to detect complex, non-random behavior patterns that do not necessarily occur in a proscribed sequence within a person. THEME™ makes no a priori assumptions about the characteristics of the behavior patterns. Rather, it allows the data to inform the patterns, providing a more accurate characterization of temporal behavior patterns, behavior complexity and escalation. THEME™ detects the complexity, frequency, and interrelationships of behavior patterns, and then quantifies these factors within person, providing a method of detecting within individual sequential and non-sequential temporal patterns (T-patterns) of related behavior clusters (chains) that are not obvious to the trained observer or identifiable by traditional sequential methods [87,88].

Initially, this pattern recognition software identifies significant (non-random) recurrences of any two behaviors, such as low intensity restlessness or vocalization. These patterns are incorporated into more complex patterns. For example, behaviors such as vocalization and restlessness at a high intensity, combined with other behaviors such as tapping and banging, interacting with a staff member, and then being left alone, can constitute a complex behavior pattern. Escalation measured in this way shows behavior clusters that include behaviors of low intensity becoming more complex and different behaviors of higher intensity. A further description of this methodology can be found in Woods et al. [78].

Utilizing this method has several advantages. It (a) is not constrained by implicit assumptions about the behaviors of interest, (b) allows the data to inform the resultant patterns, a process that is more inductive than deductive; (c) results in a more accurate characterization of behavior patterns, and (d) captures nuances in pattern escalation that cannot be detected by a trained observer or identifiable using traditional sequential methods. Once the behavior clusters have been identified and quantified, these data can be transferred into a statistical software package such as SPSS for further analysis, such as the calculation of within individual percent of patterns containing high-intensity behavior. Individual characteristics can then be compared and behavior change over time and individual patterns of response can be identified.

Limitations to the use of this software include the use of humans to collect the data using prior definitions of behaviors, issues similar to those noted previously in the paper. Questions include what the definitions of behaviors are, whether they are being observed, and what data is input and by whom? This is true if human beings are completing all of the observations but does not necessarily apply if the data are gathered by motion sensors and other types of technology such as Kinect by Microsoft, which does not require collection by human beings. Algorithms are currently being devised and tested, similar to those within THEME, to “read” the data and flag potential issues. These areas are in development and show promise for the future.

Pattern recognition software can contribute significantly to the detection and characterization of BSDs. The following are examples of what might be an expected result from different interventions.

### 8.1. Example of Pharmacological Intervention

THEME™ can detect behavioral subtlety in temporal patterns of behavioral clusters. Once these clusters are detected and quantified, pharmacological interventions can be specifically targeted and timed to alter these detected behavioral clusters of high-intensity behavior. The intervention can then be tested such that an expected outcome would be a decrease in the percentage of complex behaviors containing high-intensity behavior, which would create a more sensitive measure for determining the dosage and timing of pharmacological treatments.

### 8.2. Example of Nonpharmacological Intervention

Nursing home settings are complex environments [89], and the level of noise and crowding may increase environmental press leading to BSDs [90]. Using THEME™, the pattern of temporal behavioral clusters can be quantified and related to environmental complexity at baseline. If an intervention is applied to decrease environmental complexity, the effect could be reflected in a decrease in complex patterns of behavioral clusters. Even if the effect influences only one or two individuals with high-intensity behaviors, research shows that decreasing the BSDs of specific individuals frequently results in a generalized decrease of BSDs on the entire unit, especially in designated dementia units [91]. Interventions tailored more specifically to individuals would result in a more efficient use of resources. Once we understand who responds and the characteristics of the behavioral clusters exhibited by individuals who respond, we may be able to tailor and time effective interventions. For example, a nonpharmacological intervention may alter the complexity of BSDs by decreasing the number and intensity of behaviors that occur together, by altering the number of behavioral complexes that contain high-intensity behavior, or by altering the frequency of the behavioral complexes that occur over time. These results can decrease not only staff time and frustration but also the associated costs [92].

The ability to quantify these patterns of behavior and validate these patterns with other biological measures, such as stress hormones and temperature, is key to the development of tailored interventions. Our vision for clinical practice and management is that, by characterizing the patterns of BSD escalation, person-centered interventions [93] will be modified and timed to ameliorate BSDs and to prevent escalation and associated adverse outcomes in both PWDs and staff [78].

## 9. Conclusions

Results from studies of interventions for reducing and/or eliminating BSDs have had equivocal and somewhat disappointing results. Given the current conceptualization and strategies available for measuring outcomes, however, it is difficult to know if the problem is with the interventions or with the measurement of outcomes. The measures currently used to evaluate treatment efficacy are devised using a single indicator, a dichotomous approach that focuses on the presence or absence of specific high-intensity behaviors such as yelling and intense restlessness. These measurement strategies are based on the belief that behavioral symptoms are separate or that they may occur with one other behavior rather than the notion that behaviors cluster.

Lost in the prevailing measurement approaches is that BSDs are characterized by intensity (troublesomeness) and complexity (variability among co-occurring behaviors) as well as frequency. Temporal patterns of co-occurring behaviors or behavioral clusters are also diminished. Detecting temporal changes in behavioral clusters might be the key to detecting subtle behavior changes that herald an unrecognized treatment response or a response that suggests the need for a different treatment approach. Further information is hidden in analysis techniques that employ aggregation of data or in a priori assumptions about the nature of relationships.

The conceptual and measurement problems in studies of BSDs have been recognized for some time. Most of the currently used behavioral rating instruments were designed for cross-sectional use, and few data are available regarding their usefulness for longitudinal tracking of behaviors or their sensitivity to behavioral changes produced by specific interventions. Therefore, we reconceptualize BSDs not as single events, but as complex patterned clusters of behaviors and suggest that new analysis strategies, such as pattern recognition software, may enhance the detection of BSDs and better enable researchers to evaluate treatment outcomes.
